# Time-series transcriptomics and alternative splicing analysis of embryonic development of the asian honeybee (*Apis cerana*)

**DOI:** 10.3389/fgene.2025.1665548

**Published:** 2025-10-02

**Authors:** Xiang Ding, Zukai Liu, Linsen Ou, Zhihui Wang, Qian Xu, Runlang Su

**Affiliations:** ^1^ School of Mechanical and Electrical Information, Yiwu Industrial and Commercial College, Jinhua, China; ^2^ University of Chinese Academy of Sciences, Beijing, China; ^3^ Fujian Port & Waterway Investigation and Design Institute Limited, Fuzhou, China

**Keywords:** *Apis cerana*, embryonic development, alternative splicing, time-seriestranscriptome, differentially expressed genes (DEGs)

## Abstract

Embryonic development in honeybees is a critical stage that shapes the formation of organs and structures in adult bees. Although there has been substantial progress in transcriptome studies on honeybee embryogenesis, time-series transcriptomic and alternative splicing data in the embryonic development of the Asian honeybee (*Apis cerana*) remain limited. In this study, we conducted an in-depth analysis of RNA-seq data from public databases to examine the transcriptomic profiles at three key developmental stages of *A*. *cerana* embryos (24 h, 48 h, and 72 h), uncovering the dynamic changes in gene expression and alternative splicing across these stages. Results showed that the number of differentially expressed genes and alternative splicing events peaked at 24 and 48 h and then gradually decreased. Time-series transcriptomic analysis further identified key physiological and biochemical processes at these stages, reflecting a progression from foundational metabolism and cellular structure construction in the early stages to cell differentiation and organogenesis in the middle stage, and finally to functional structure refinement and behavioral trait formation in the later stage. Notably, our study highlighted the central role of alternative splicing and gene expression in driving key physiological and morphological changes during embryogenesis. We identified multiple key genes, including DMRT family genes, the Maelstrom (Mael) gene, and highly GO-enriched genes such as *Dll*, *CaMKII*, and *Cnn*. These genes not only play essential roles in structural formation but also support neurodevelopment and the emergence of complex behavioral patterns in adult bees. Gene expression and splicing patterns at different developmental stages provide new insights, revealing the early foundational mechanisms underlying limb development, behavior and memory, sensory organ development, and neural plasticity in honeybees.

## 1 Introduction

The Asian honeybee (*Apis cerana*) is one of the primary bee species widely cultivated in East Asia, with a rich beekeeping tradition in China, Japan, and India, contributing significantly to the beekeeping industry. Unlike Western honeybees, *A*. *cerana* has an extremely high sensitivity to scents, allowing it to make more effective use of dispersed nectar sources, making it well-suited to gather from a variety of nectar plants, whereas Western honeybees tend to prefer large-scale, single nectar sources. Additionally, *A*. *cerana* demonstrates outstanding disease resistance and adaptability, showing resilience to cold temperatures, lower feed requirements, and a longer foraging period (Alamancos et al., 2015). These characteristics give *A*. *cerana* a unique advantage in diverse ecological environments, contributing to sustainable agricultural and ecological development.

The development of honeybees consists of four life cycle stages: embryo, larva, pupa, and adult (Cantalapiedra et al., 2021). Embryonic development, the earliest and most critical stage, is precisely regulated by environmental factors and intracellular signals (Chen et al., 2016). During this stage, the rudimentary organs of the adult bee are gradualy formed, making the bee embryo an ideal model for genetic modification (Chen et al., 2011).

With the decreasing costs of long-read sequencing and advancements in sequencing technology, high-quality genome assembly for non-model organisms has become achievable. The first high-quality genome of the Italian honeybee was published in 2019 (Chen et al., 2019), followed by the successful assembly of the first high-quality *A*. *cerana* genome in 2020 (Alamancos et al., 2015). Improved reference genome assembly has provided multiple advantages for RNA-seq data analysis, including higher efficiency in read alignment, increased accuracy in identifying differentially expressed genes, and enhanced precision in detecting alternatively spliced transcripts. These benefits result from the high-quality genome’s reduction of the effects from single nucleotide polymorphisms (SNPs) and annotation inconsistencies, thereby improving the overall accuracy and reliability of transcriptomic studies (Cridge et al., 2017).

Proteomics and transcriptomics are key research areas in studying honeybee embryonic development. Previous studies have found distinct phosphorylation patterns in worker and drone bees during their respective developmental stages, with worker bee embryos showing significantly enhanced phosphorylation signals during germ layer formation and organogenesis (Chen et al., 2011). Given the 3-day duration of the honeybee embryonic development period, research has primarily focused on sampling at 24, 48, and 72 h to capture critical developmental stages (Chen et al., 2016; Chen et al., 2011; Fang et al., 2015; Garcia-Blanco, 2005). These studies lay an important foundation for understanding the molecular regulatory mechanisms during honeybee embryonic development.

Alternative splicing (AS) is a crucial focus in transcriptomics research, greatly enhancing protein diversity by enabling plants and animals to produce different protein isoforms to adapt to environmental changes. AS expands the coding capacity of genes, adding unparalleled complexity to transcriptomes and proteomes. This complexity varies across developmental stages, tissues, and environmental perturbations, significantly impacting gene expression regulation (Haas et al., 2013). Studies have shown that AS is prevalent across various species, correlating with traits like drought resistance (Heuer et al., 1995), agronomic traits (Hu et al., 2018), and heat resistance (Kim et al., 2015) in plants. Likewise, in animals such as brine shrimp, birds, zebrafish, and sea cucumbers, AS is closely linked to sex regulation (Li et al., 2009; Li and Kaufman, 1996; Liao et al., 2014; Lin et al., 2022). However, the spatiotemporal dynamics of AS during honeybee embryogenesis remain largely unexplored.

Through RNA-Seq analysis, time-series transcriptome analysis, and alternative splicing analysis, we identified stage-specific gene expression patterns during honeybee embryonic development. Our findings reveal several non-coding genes that may play key roles in embryonic differentiation and identify three core genes driving complex developmental processes. These core genes are pivotal in specific alternative splicing events, contributing to the regulation of limb development, photoreception, and memory function. Overall, these data highlight the diversity and complexity of AS in honeybee embryogenesis.

In summary, our research deepens the understanding of honeybee embryonic development and its molecular mechanisms, offering a comprehensive view of the physiological and biochemical processes involved. Our findings provide valuable candidate genes for future developmental research and hold significant potential for applications.

## 2 Materials and methods

### 2.1 Source of sequence data

The analyses in this study are based on publicly available sequence data from the NCBI GenBank database under accession number SRP131292 (https://www.ncbi.nlm.nih.gov/sra/?term=SRP131292) (Chen et al., 2016). The datasets used and analyzed were obtained from this publication and can be requested from the corresponding author of the original study upon reasonable request.

### 2.2 Mapping and quality control of RNA-seq data

Before alignment, the raw data were quality-checked and cleaned using FastQC (http://www.bioinformatics.babraham.ac.uk/projects/fastqc/). Specifically, we used the default parameters of FastQC to assess quality scores (Q values), GC content, and the proportion of N bases in the reads. The filtered reads were then aligned to the reference genome (GCA_029169275.1) using Hisat2 (v2.2.1) with the following parameters: -max-intronlen 10,000 (maximum intron length set to 10,000 bp), --dta (optimization for downstream transcript assembly), and--phred33 (Phred+33 quality score encoding) (Wang et al., 2021a). The resulting SAM files were converted to BAM format, sorted, and indexed with Samtools (v1.6) (Ma et al., 2022). Quantification of aligned reads was performed using FeatureCounts (Patro et al., 2017). Finally, differential expression analysis was conducted using Trinity (v2.15.1) with the DESeq2 algorithm (Rogers et al., 2021).

### 2.3 GO and KEGG analysis

Using emapper. py (v2.1.10) with the eggNOG database (Roth et al., 2023), GO and KEGG annotations were performed on the protein sequence of the longest csd sequence in the reference genome of *A*. *cerana*. The annotated file was then converted into org. db format to facilitate GO and KEGG enrichment analysis using the clusterProfiler (v4.0) package in R (Setton and Sharma, 2018).

### 2.4 Temporal clustering analysis of genes

Time-series transcriptome analysis was conducted on significant genes in the expression matrix using the ClusterGVis package in R (https://github.com/junjunlabClusterGVis). By clustering similar genes, gene modules in different clusters were analyzed to understand gene expression patterns across various embryonic stages. The ClusterGVis package integrates fuzzy C-means (Mfuzz) and K-means clustering algorithms, allowing for efficient clustering analysis of gene expression matrices and providing insights into gene expression trends at each time point.

### 2.5 Alternative splicing analysis workflow

For alternative splicing analysis, filtered sequencing reads were aligned to the reference genome (GCA_029169275.1) using the Salmon software, which provides a fast and accurate tool for quantifying transcript expression. Salmon aligns the reads pseudo-selectively, allowing for quantification without the need to generate large alignment files. This approach is efficient in handling complex transcriptomes, especially when exploring alternative splicing events across multiple samples (Slabaugh et al., 2019). Next, alternative splicing events were identified using the SUPPA (Simulation of Uncertainty and Prediction of Alternative splicing) tool (Song et al., 2020; Tao et al., 2021). SUPPA leverages transcript-level quantification data from Salmon and annotation files (e.g., GTF format) to generate alternative splicing events, such as exon skipping, alternative 5′ and 3′ splice sites, and intron retention. By applying SUPPA’s splicing event generation functionality (suppa.py), the annotated files were processed to identify splicing variations across conditions, providing insights into how alternative splicing patterns may vary between developmental stages or treatment groups (Song et al., 2020; Tao et al., 2021).

## 3 Results

### 3.1 Overall RNA-Seq results and quality control

The Illumina RNA sequencing generated an average of 86.89 million (M) raw reads per sample. After filtering, an average of 86.59 million clean reads per sample was retained for subsequent transcriptome analysis. The average quality scores for clean reads showed Q20 and Q30 ratios of 100%, indicating high quality of the obtained clean reads. Additionally, the average GC content was 38.24% (Supplementary Table S1). We used HISAT2 software to perform alignment analysis with the reference genome. The sliding window density analysis showed that over 80% of the transcripts were uniquely aligned to the reference genome, with an average alignment rate of 83.33% across nine samples. Only 15.66% of the reads (7.97 million) failed to align to the genome (Supplementary Table S2).

### 3.2 Hierarchical clustering and principal component analysis of samples

**FIGURE 1:**
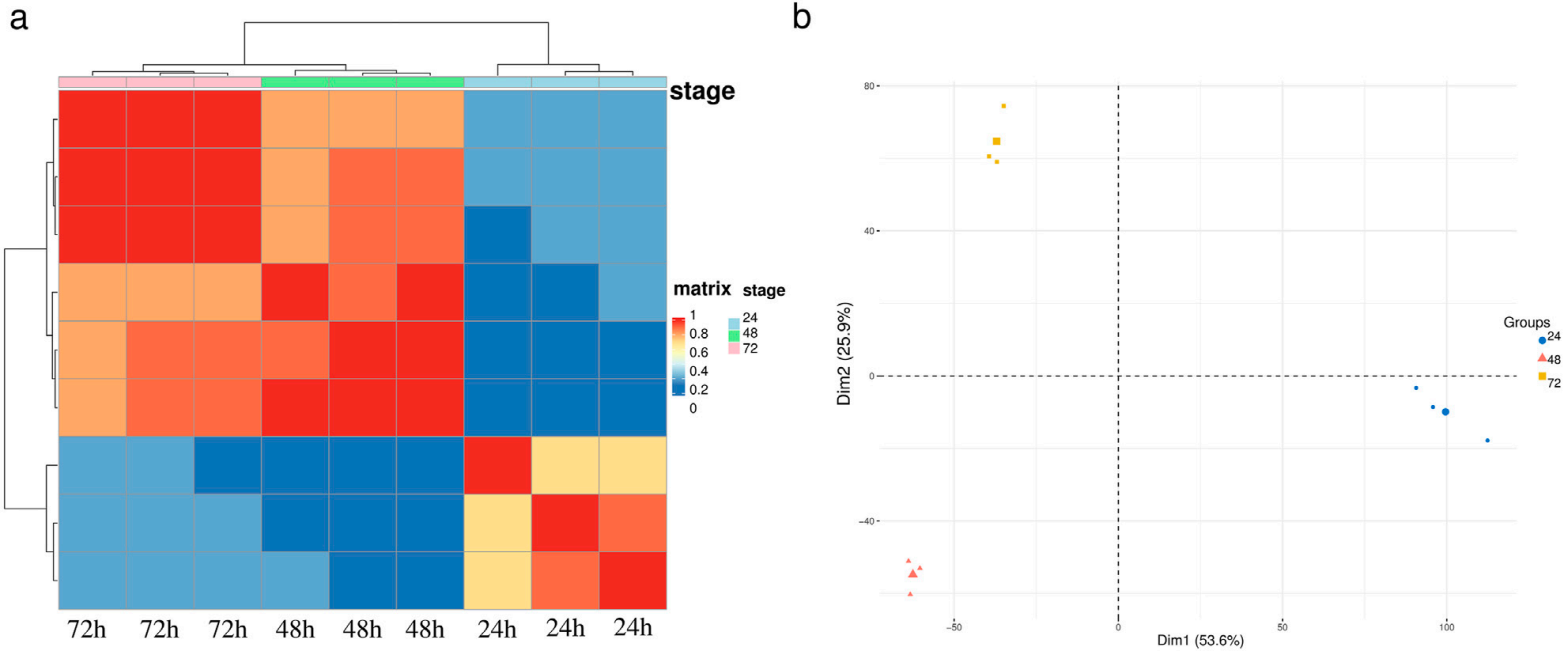
Sample clustering for embryos of *A*. *cerana*. **(a)** Heat map and hierarchical clustering of the embryo samples using the whole transcriptome data. **(b)** Principal component analysis (PCA) of the embryo samples using the whole transcriptome data.

To assess the consistency of sample collection and explore the transcriptomic relationships among embryos at 24, 48, and 72 h, we conducted hierarchical clustering and principal component analysis (PCA) on the global gene expression data of these samples. These two analytical methods summarize the similarities and differences among the samples. Prior to these analyses, we normalized the gene expression levels using the regularized log transformation method implemented in the limma package. The results showed that the 48-hour and 72-hour samples were more similar to each other, while both time points exhibited significant differences compared to the 24-hour samples, consistent with previous studies (Figure 1a). In the PCA plot (Figure 1b), embryo samples collected at the same time point clustered together, further validating the results of the hierarchical clustering analysis. Notably, the 24-hour samples were distinctly separated from the 48-hour and 72-hour samples along the first principal component (PC1), indicating that PC1 primarily reflects the gene expression differences between the 24-hour stage and subsequent developmental stages. Meanwhile, the 48-hour and 72-hour samples were differentiated along the second principal component (PC2), suggesting additional transcriptomic differences between these two time points. Moreover, we found that the Apd−2 and HEX70b gene had high loadings on both PC1 and PC2 (Supplementary Figure S1), indicating its significant role in distinguishing samples from different developmental stages. Both analyses demonstrate that gene expression at 24 h of embryonic development was significantly different from the subsequent two stages, suggesting that the most substantial changes and regulation in gene expression occur between 24 and 48 h.

### 3.3 Analysis of differentially expressed genes (DEGs)

**FIGURE 2:**
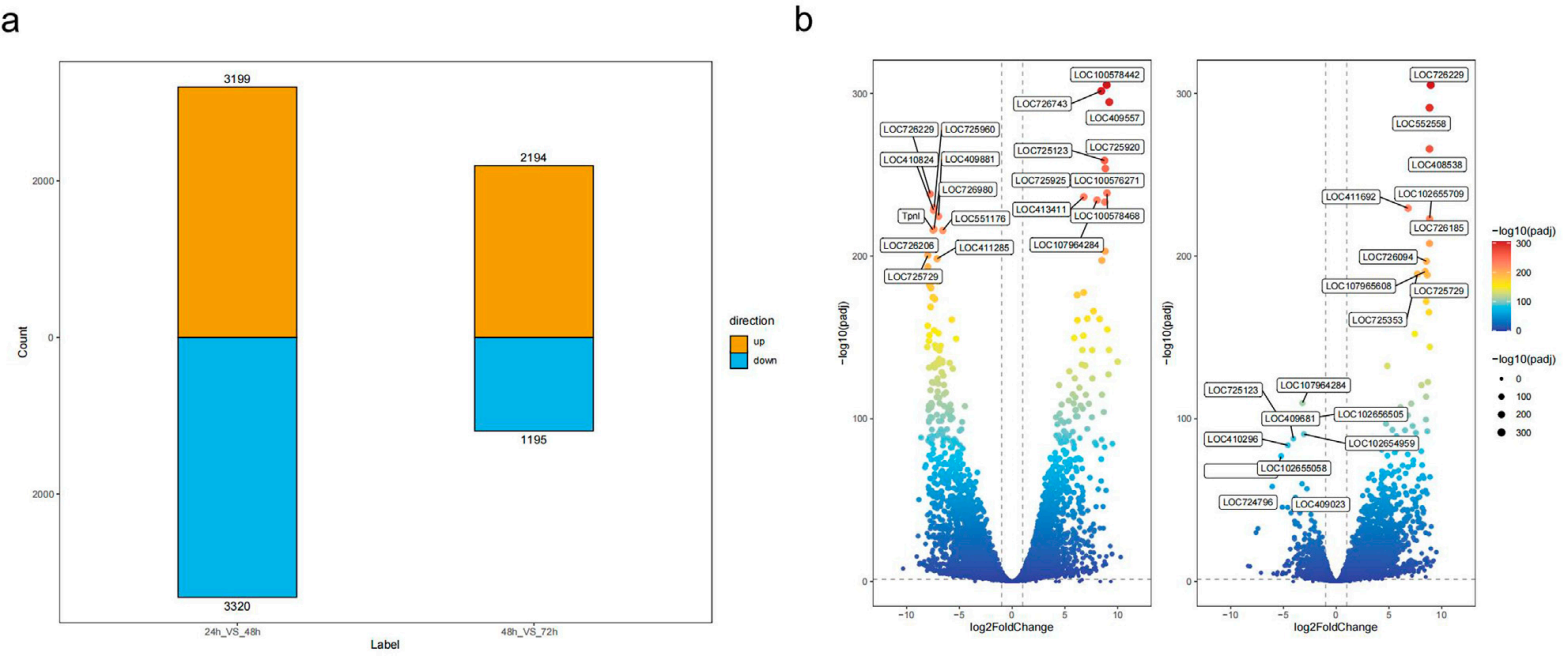
Differential Gene Expression Analysis. **(a)** Statistical summary of differentially expressed genes across the developmental stages, showing the count of upregulated (orange) and downregulated (blue) genes in the comparisons between 24 h vs. 48 h and 48 h vs. 72 h. **(b)** Volcano plots illustrating the differential expression of genes across three embryonic developmental stages. The left plot shows the comparison between 24 h vs. 48 h, and the right plot shows the comparison between 48 h vs. 72 h. Genes with |log2FoldChange| > 1 and -log10 (padj) > 0.05 are displayed, with labels indicating the most significantly differentially expressed genes. Gene color represents the degree of significance based on the -log10 (padj) values, and the size of the points reflects the fold change.

The volcano plots (Figure 2b) visually illustrate the overall trends in gene expression levels across stages. Each point represents a gene, with the horizontal axis showing the logarithmic fold change in gene expression (log2 Fold Change) and the vertical axis showing the negative logarithm of the significance level (−log10 p-value). Significantly upregulated genes were distributed in the upper right of the plot, while significantly downregulated genes appear in the upper left. It is evident that the number of differentially expressed genes is highest during the 24-hour to 48-hour stage; the volcano plot displayed a more dispersed pattern, indicating the most dramatic changes in gene expression occur at this stage. This was consistent with the previous hierarchical clustering and principal component analysis results, further confirming that the period from 24 to 48 h was a critical turning point in embryonic development, during which significant reprogramming of gene expression occurs.

In contrast, from 48 h to 72 h, the number of significantly differentially expressed genes in the volcano plot decreases markedly, and the plot becomes more concentrated. This suggested that the degree of gene expression changes was reduced during this stage, and the embryonic development process enters a relatively stable period.

The genes showing significant differential expression in these volcano plots may be closely related to important physiological and biochemical processes, morphological formation, and functional differentiation at each embryonic stage. Upregulated genes may be involved in cell proliferation, differentiation, and the acquisition of specific functions, while downregulated genes may be associated with the regulation of the cell cycle and adjustments in metabolic activities. Further functional annotation and pathway enrichment analyses of these differentially expressed genes will help reveal their specific roles and regulatory mechanisms in embryonic development, providing important insights into the molecular basis of embryogenesis.

### 3.4 Temporal transcriptome analysis of embryonic development using k-means clustering

**FIGURE 3:**
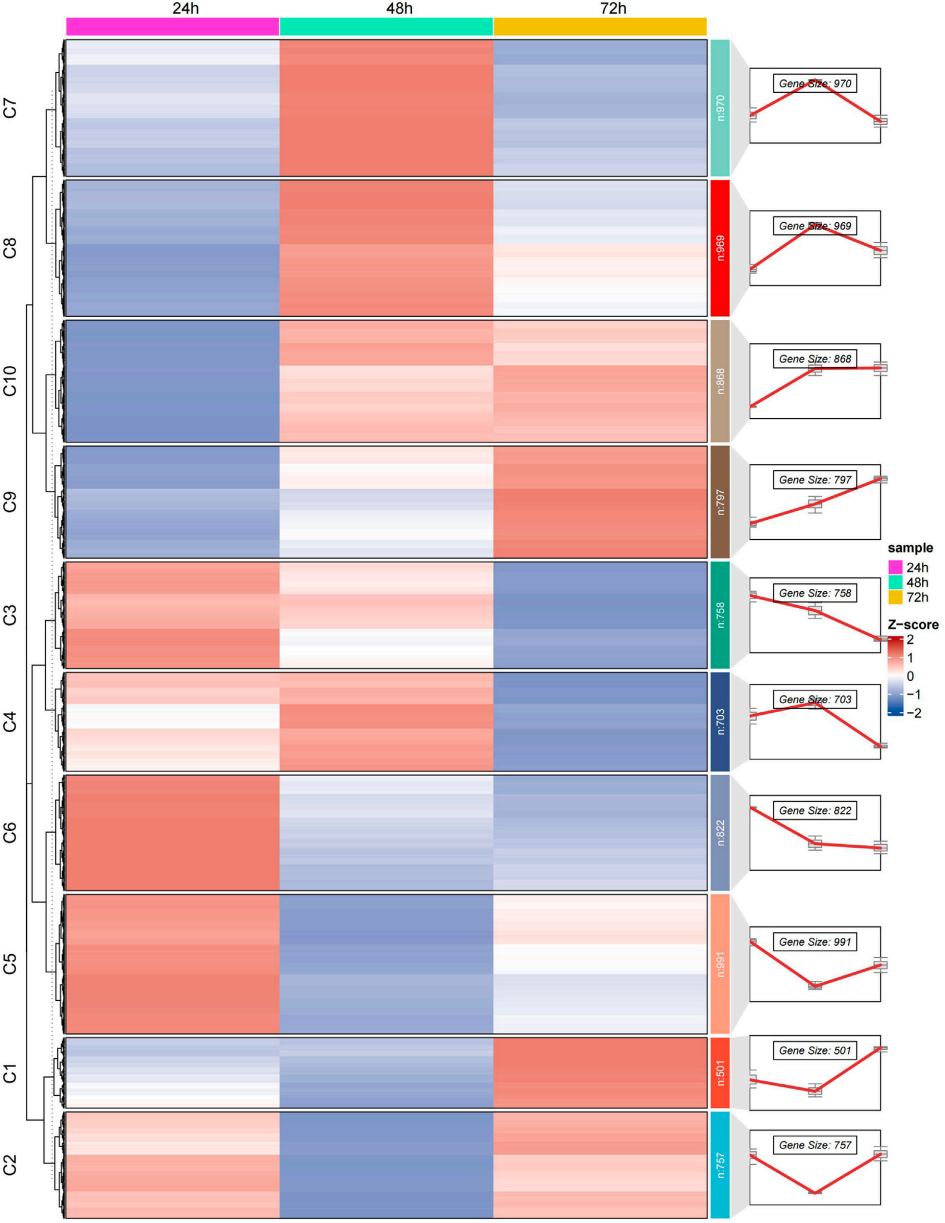
Illustrates the dynamic changes in gene expression during the embryonic development of the Asian honeybee (*A*. *cerana*). Using k-means clustering analysis, we divided the gene expression patterns into 10 clusters. The heatmap on the left side of the figure shows the gene expression levels at various developmental stages, while theline plots on the right depict the trends in gene expression at three key time points during embryonic development (24 h, 48 h, and 72 h). The clusters are labeled from C1 to C10.

To further explore the dynamic changes in gene expression during embryonic development, we employed a k-means clustering algorithm to partition 9,492 genes into 10 clusters based on the similarity of their expression patterns. Detailed analysis of these clusters revealed significant enrichment of genes at specific developmental stages (Figure 3).

At the 24-hour embryonic stage, genes in clusters C3, C5, and C6 exhibited significant enrichment. The associated Gene Ontology (GO) terms mainly include fatty acid derivative metabolic processes, biosynthetic processes of organic acids and carboxylic acids, key complexes related to transcription and translation, mRNA metabolism and splicing, and the metabolic processing of rRNA and ncRNA (Supplementary Tables S3, S4, S5). These results indicated that at the 24-hour stage, gene expression primarily involves fundamental metabolic processes and the establishment of gene expression machinery, emphasizing the construction of basic cellular components and functions required for early development.

Overall, these genes play crucial roles in multiple key processes and functions of cell biology and developmental biology throughout various stages of embryonic development. As development progresses into the 48-hour stage, genes in clusters C7 and C8 showed high expression levels. The related GO terms were concentrated on the positive regulation of biological and cellular processes, post-embryonic development, larval or pupal development, appendage morphogenesis, and RNA synthesis, modification, and processing (Supplementary Tables S6, S7). This suggested that at the 48-hour stage, genes were involved in developmental programs that initiated morphological changes and cell differentiation, promoting the formation of specific structures while continuing RNA processing activities. Further, at the 72-hour stage, genes in clusters C9 and C10 become predominant. The associated GO terms mainly pertain to protein synthesis processes, the structure and function of ribosomes, appendage development and morphogenesis (including imaginal disc-derived appendage development, appendage morphogenesis, and leg disc development), and specific molecular binding and enzymatic activities (such as signal receptor binding, heme binding, tetrapyrrole binding, and nucleotide monophosphate kinase activity) (Supplementary Tables S8, S9).

These resulted reflect that at the 72-hour stage, gene expression focuses on protein synthesis and the role of ribosomes as the core machinery for protein production, promoting further development of appendages and demonstrating diverse functions in molecular binding and catalysis, indicating an advanced stage of tissue formation and functional specialization.

### 3.5 Alternative splicing and its role in gene regulation during early embryonic development

**FIGURE 4:**
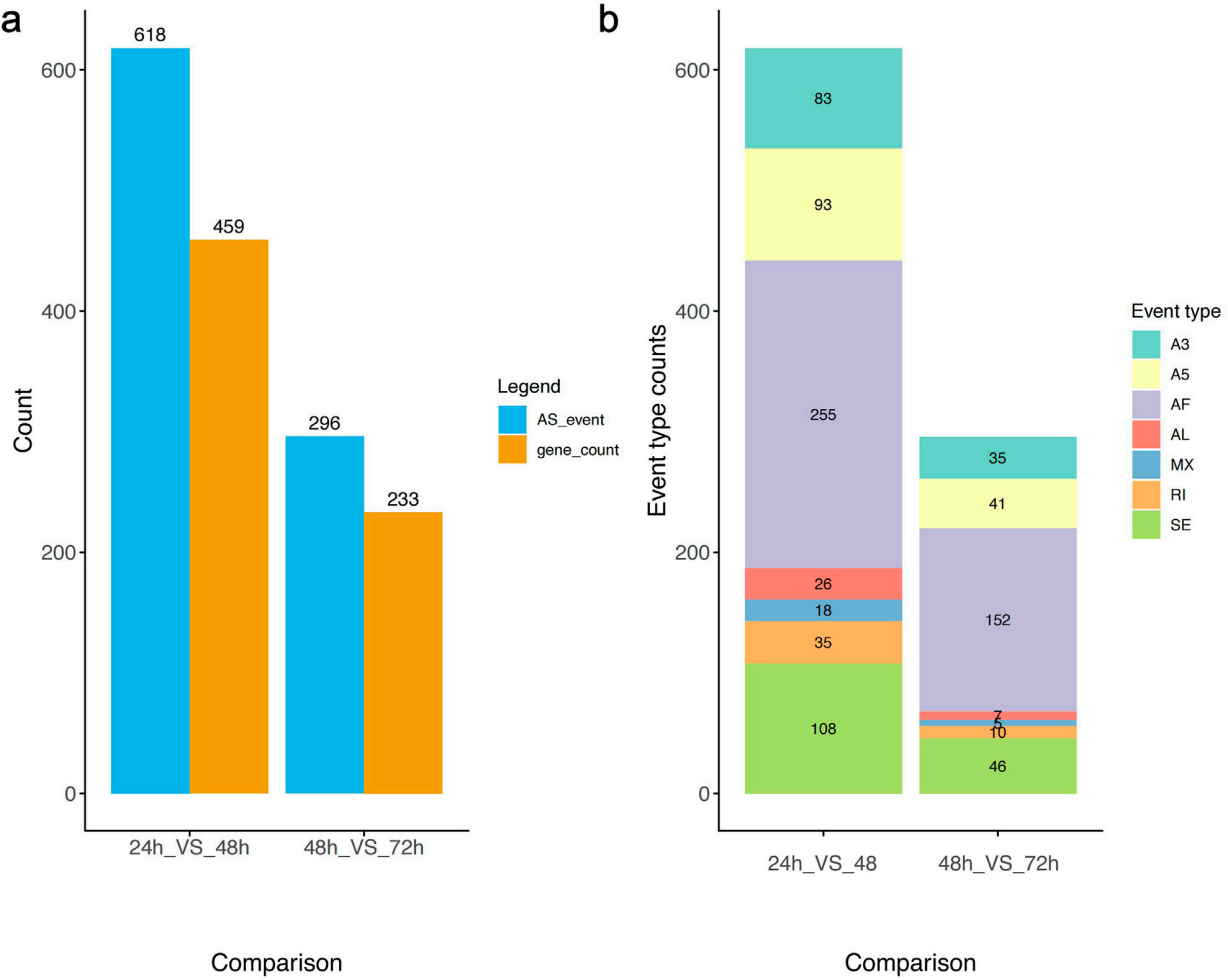
Differential isoforms expression analysis. **(a)** Variable splicing events and the corresponding gene count in three time points of *A*. *cerana* embryonic development. **(b)** Graph showing the number of seven types of alternative splicing events across three time points of *A*. *cerana* embryonic development.

Alternative splicing analysis validated the expression levels of different gene transcripts at three time points during embryonic development. The results showed that the numbers of alternatively spliced genes and splicing events at these three time points significantly overlapped with the distribution of differentially expressed genes. During the critical developmental stage from 24 to 48 h, the counts of all seven types of splicing events reached their highest levels, with Alternative First Exon (AF) events being the most abundant, totaling 255 (Figures 4a,b).

The high frequency of AF events may be related to the complexity and diversity of gene expression regulation in the early stages of embryonic development. Between 24 and 48 h a pivotal developmental period embryonic cells undergo rapid proliferation and differentiation, necessitating precise and flexible mechanisms of gene expression regulation. AF events, by selecting different initial exons, can produce protein isoforms with varying N-terminal structures, thereby influencing protein localization, function, and interactions with other molecules. This mechanism allowed the same gene to perform diverse functions in different cell types or developmental stages.

### 3.6 GO enrichment in alternative splicing events

**FIGURE 5:**
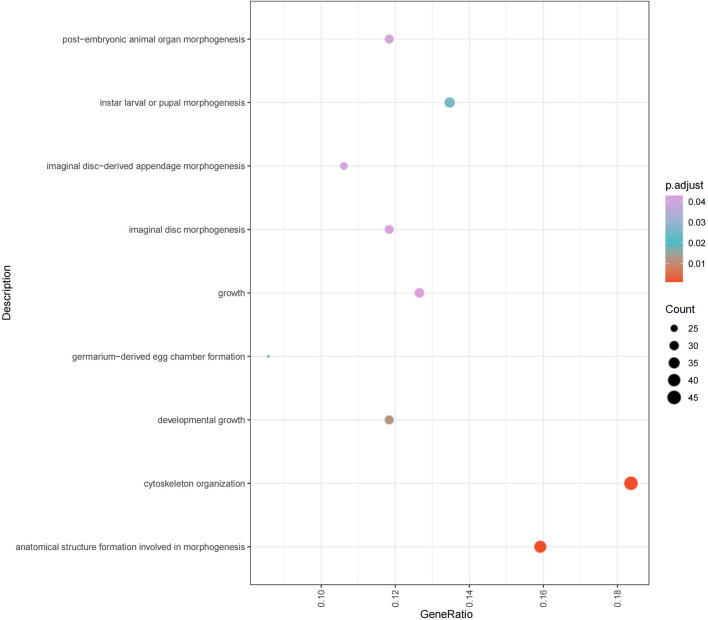
Embryonic development alternative splicing GO enrichment chart (p.adjust<0.05).

The results of GO enrichment analysis (Figure 5) indicate that significantly differentially spliced transcripts in embryos are primarily involved in biological processes such as morphogenesis, growth, cytoskeleton organization, and the formation of specific organs and appendage structures. Specifically, these functions encompass biological processes such as cytoskeleton organization, morphogenesis, neuromuscular junction formation, axon development, cell polarity, protein polymerization, cell junction organization, organ development, and cell structure formation (Supplementary Table S10). These processes include core functions such as cell differentiation, signal transduction, structural assembly, and cell-cell junctions, revealing the role of complex regulatory networks in maintaining and modulating the morphology and function of cells and tissues during development.

These findings suggest that alternative splicing events may play an essential role in regulating the formation and maintenance of specific cellular structures at critical stages of embryonic development. For instance, the dynamic regulation of cytoskeleton and cell junctions is crucial for maintaining cell morphology, differentiation, and migration, while regulation of protein polymerization and axon development may be pivotal in the formation of the nervous system. Additionally, these differentially spliced transcripts may have significant roles in the morphogenesis and physiological functions of specific organs and appendages (e.g., muscle and nervous system), highlighting the highly refined gene expression regulatory mechanisms during embryonic development. These insights provide important clues for further exploration of how alternative splicing mediates functional diversity of genes and precise regulation in specific developmental processes.

### 3.7 Alternative splicing dynamics of key genes Dll, CaMKII, and LOC408563 during embryonic development

**FIGURE 6:**
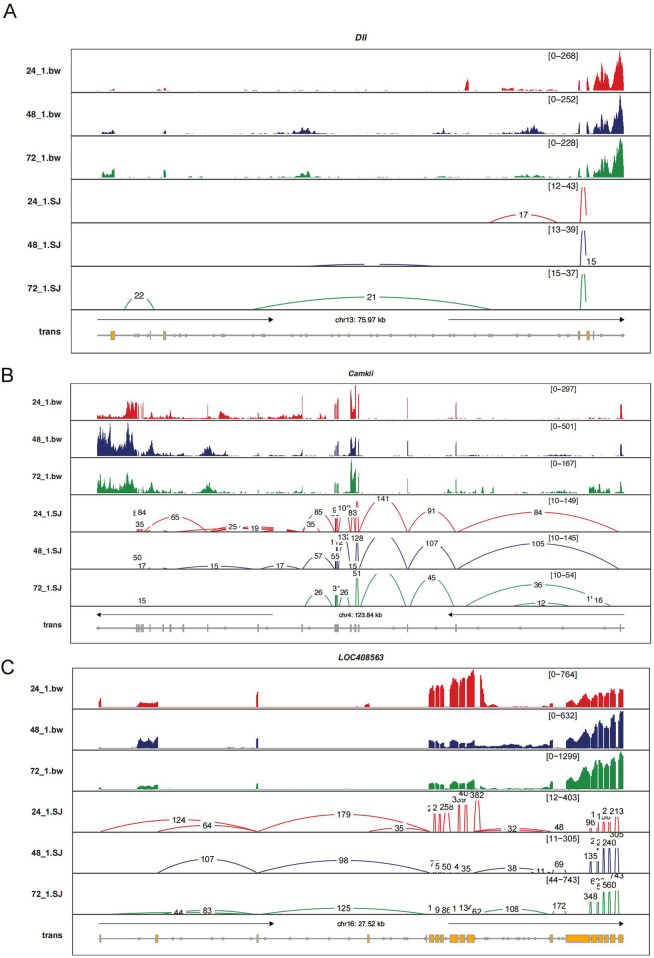
Sashimi Plot Illustrating Levels of Intron Retention and Exon Usage for Three Genes (*Dll, CaMKII, LOC408563*). **(a)** Gene *Dll*, **(b)** *CaMKII*, and **(c)** *LOC408563*. In the legend, yellow represents exons, with red, blue, and green lines corresponding to time points 24 h, 48 h, and 72 h, respectively. The connected line segments represent the gene segments that are linked together. (The data represented here only includes contigs with a count greater than 10, as we filtered out contigs with lower counts to focus on more reliable and significant splicing events.).

To investigate the key genes involved in alternative splicing events during embryonic development, we selected three genes with the highest connectivity in GO enrichment analysis: *Dll*, *CaMKII*, and *LOC408563*. Our results showed that the *Dll* gene exhibited exon-skipping events at both the 24-h and 48-h time points, with each time point featuring unique exon skipping events. Additionally, at 72 h, an additional exon skipping event was observed compared to the 48-h time point (Figure 6).

In the *CaMKII* gene, compared to 48 h, the 24-hour time point had two additional exon skipping events and one intron retention event. At 72 h, *CaMKII* showed one unique intron retention event but had three fewer exon skipping events and one fewer intron retention event. The *LOC408563* gene exhibited one more exon skipping event and one more intron retention event at 24 h compared to 48 h. Conversely, at 48 h compared to 24 h, it had one unique intron retention event. Compared to 72 h, the 48-hour time point had two more exon skipping and one more intron retention event, while at 72 h, a unique intron retention event was present (Figure 6).

The changes in these splicing events at different developmental stages may suggest distinct roles for these genes in regulating embryonic development. Time-specific splicing patterns likely relate to the precise regulatory requirements of gene expression, with such dynamic splicing mechanisms enabling the production of different protein isoforms to meet the varying functional needs of cells during development. Thus, these alternative splicing events could represent an important aspect of the regulatory complexity in embryonic development.

**FIGURE 7:**
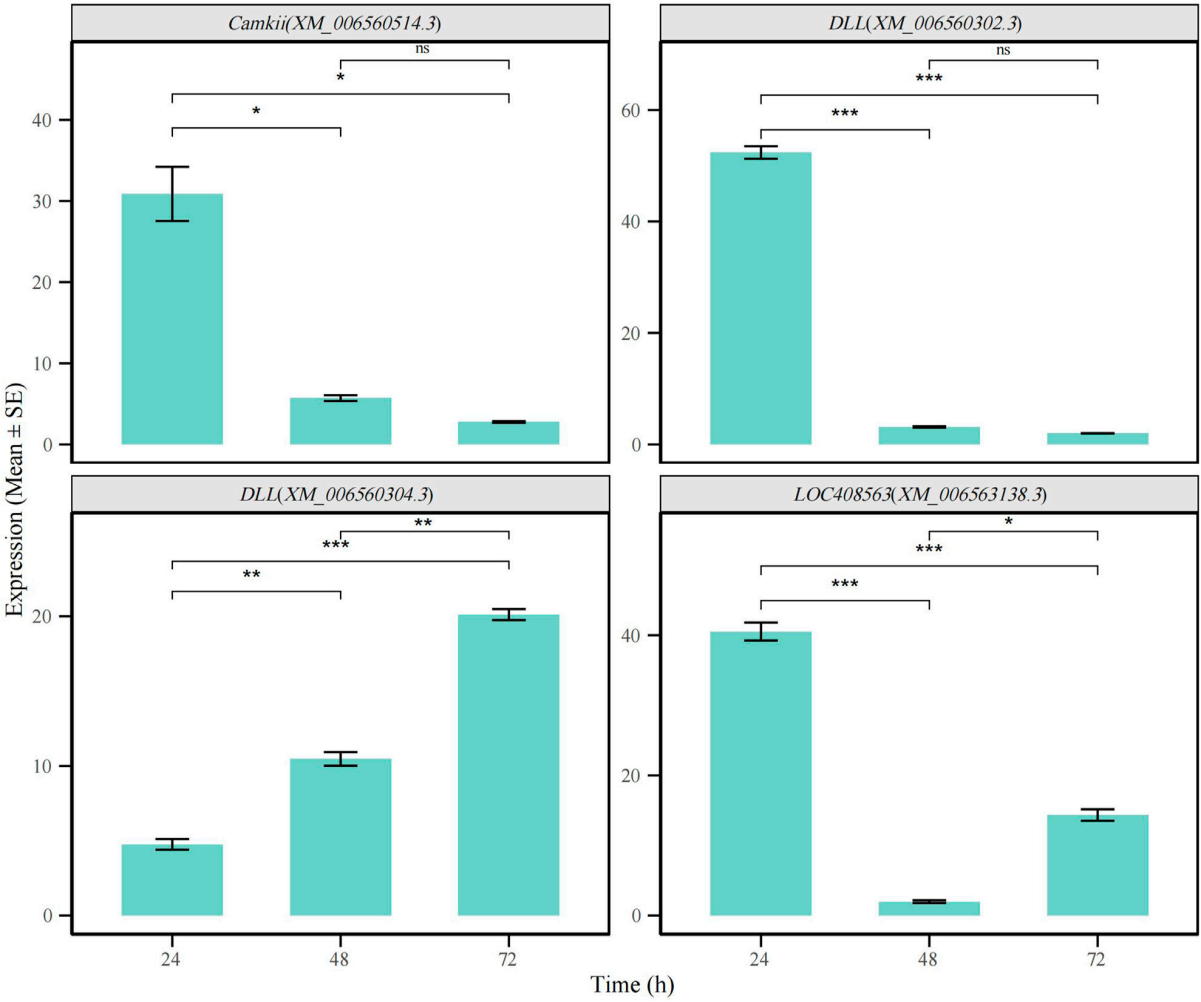
qRT-PCR validation of alternative splicing events in *CaMKII*, *Dll*, and *LOC408563* genes across different time points (t-test,p < 0.05). (*p < 0.05, **p < 0.01, ***p < 0.001).

To further validate the reliability of our public database-based analysis results, we performed independent verification of the identified differentially spliced genes using quantitative real-time PCR (qRT-PCR). Specifically, we selected three representative genes, *Dll* (*XM_006560304, XM_006560302.3*), *CaMKII* (*XM_00650514.3*), and *LOC408563* (*XM_006563138.3*), and validated the transcripts corresponding to the relatively unique splicing events at the 24-hour, 48-hour, and 72-hour time points. The results demonstrated that the alternative splicing patterns of these three genes were highly consistent with those predicted by our bioinformatics analysis, confirming the accuracy and reliability of our analytical pipeline (Figure 7; Supplementary Table S11).

## 4 Discussion

To investigate the gene expression levels and alternative splicing changes in Asian domestic honeybee embryos against a high-quality genome background, we conducted RNA-seq analysis on embryos at three developmental time points: 24 h, 48 h, and 72 h. Compared with previous studies (Chen et al., 2016), the high-quality genome significantly enhanced alignment efficiency and the number and accuracy of identified differentially expressed genes (Figure 2a; Supplementary Table S2). Additionally, principal component analysis (PCA) and clustering heatmap analysis further validated the reliability and reproducibility of our data, with conclusions consistent with earlier research, including the expression of photoreception genes (Figures 1a,b).

Through volcano plot analysis, we identified genes with significant differential expression between different time points. Notably, *LOC726743* exhibited significantly high expression during the 24 h–48 h period. This gene belongs to the DMRT transcription factor family, known in *Drosophila* with the *dsx* gene as a representative that regulates sex-specific behaviors (Trincado et al., 2018). This finding suggests that differential expression of DMRT family genes may have been activated early in honeybee embryonic development, participating in sex differentiation and the formation of worker bee-specific behavioral patterns.

Furthermore, we discovered that the gene *LOC409557* (encoding the Maelstrom protein) showed significantly high expression during the 24 h–48 h period. The Maelstrom protein plays a crucial role in spermatogenesis in silkworms, and its high expression in honeybees may imply specific functions in worker bee development, particularly in regulating genome stability and cell differentiation (Viet et al., 2022). We also identified unknown function genes with significantly high expression at different time points, such as *LOC100578442*, *LOC726229*, and *LOC552558*.

To gain deeper insights into the dynamic changes in gene expression during embryonic development, we performed time-series transcriptome analysis. The results showed that honeybee embryonic development gradually transitions from basic metabolism and gene expression activities to advanced tissue formation and functional specialization (Figure 3). At the 24 h stage, non-coding RNA metabolic regulation plays a critical role in preparing for subsequent cell differentiation. By the 48 h stage, gene expression shifts toward processes of cell specialization and morphological changes, initiating the formation of appendages and differentiated cell structures. At the 72 h stage, gene expression focuses on protein synthesis and ribosomal activity, supporting further structural and functional diversity.

We also found that the distribution of alternative splicing events highly overlaps with differentially expressed genes, both peaking in number during the 24 h–48 h period (Figures 4a,b), indicating more active gene alternative splicing in early embryonic development. Among these, alternative first exon (AF) events occur frequently at this stage, possibly because early development requires highly flexible gene expression regulatory mechanisms to meet the demands of rapid cell proliferation and differentiation (Wallberg et al., 2019).

Through GO enrichment analysis of alternative splicing events, we found they play significant roles in morphogenesis, organ development, cytoskeleton remodeling, and growth regulation (Figure 5). This suggests that alternative splicing supports the structural and functional needs of embryos during rapid development by regulating the expression of genes related to cell structure and development.

Further analysis revealed three high-frequency genes highly associated with multiple GO terms: *Dll*, *CaMKII*, and *LOC408563* (Figures 6a–c). The *Dll* gene plays an essential role in arthropod development, regulating the formation of mouthparts, limbs, spider spinnerets, and other appendage structures (Wang X. et al., 2023; Wang Y. et al., 2023). Based on these findings, we speculate that in honeybees, the *Dll* gene not only participates in limb development but may also play a key role in the formation of mouthparts and stingers. The *CaMKII* gene is also crucial in nervous system development, especially in learning and memory processes. The alternative splicing of the *CaMKII* gene may provide a molecular basis for honeybee nervous system development, contributing to the complex behavioral patterns and environmental adaptability of workers (Wang et al., 2020; Wu et al., 2021). For the *LOC408563* gene, homology analysis indicates it corresponds to the *Cnn* gene. In *Drosophila*, the *Cnn* gene primarily regulates photoreception, cytoskeletal stability, maintenance of cell polarity and morphology, and plays a pivotal role in cell division and nervous system development (Zalcman et al., 2018; Zhang et al., 2024). Besides, based on previous studies, sex differentiation in both *Apis cerana* and *Apis mellifera* is regulated by the *Csd* gene, which controls the expression of the sex-specific gene *Fem* through splicing, and further affects the sex-specific splicing of the downstream gene *Dsx*. Therefore, sex differentiation in *Apis cerana* and *Apis mellifera* follows similar pathways and genes (Su et al., 2025; Wang X. et al., 2021). This aligns with the conclusions from the GO enrichment analysis in the original article (Chen et al., 2016), suggesting that gene expression and alternative splicing in the early embryonic stage may collectively influence photoreception and neural development. The alternative splicing of the *Cnn* gene not only ensures the normal progression of the cell cycle but also supports the development and functional specialization of specific tissues, particularly in nervous system development, potentially laying the foundation for the subsequent behavioral complexity of honeybees.

In conclusion, the alternative splicing of high-frequency genes plays a pivotal role in the formation of key structures in early honeybee embryos and the precise regulation of the nervous system and other physiological features. Through gene alternative splicing, honeybee embryos can adapt to different developmental needs, gradually establishing a complex behavioral foundation and physiological characteristics. These results provide important evidence for further exploring the molecular mechanisms of honeybee behavior and functional differentiation, and also reveal the significance of alternative splicing in the embryonic development process.

## 5 Conclusion

Our research utilized high-quality genome data and further mined transcriptome resources from public databases, enhancing the analytical precision of gene expression and alternative splicing in Asian domestic honeybee (*A. cerana*) embryos. Compared with the original research that only used draft genomes for alignment and focused solely on gene expression, we further explored the dynamic changes of alternative splicing events and time-series transcriptomes. The identified differentially expressed genes and alternative splicing events, especially key genes such as the DMRT family, Mael gene, *Dll*, *CaMKII*, and *Cnn*, may play important roles in honeybee embryonic development, sex differentiation, and the formation of complex behaviors.

## Data Availability

The datasets presented in this study can be found in online repositories. The names of the repository/repositories and accession number(s) can be found below: https://www.ncbi.nlm.nih.gov/genbank/, SRP131292.
