# Supplementary figures and images for: Time-series transcriptomics and alternative splicing analysis of embryonic development of the asian honeybee (Apis cerana)
Source: Front Genet. 2025 Oct 2;16:1665548. doi: 10.3389/fgene.2025.1665548 (PMC12528113; doi:10.3389/fgene.2025.1665548)

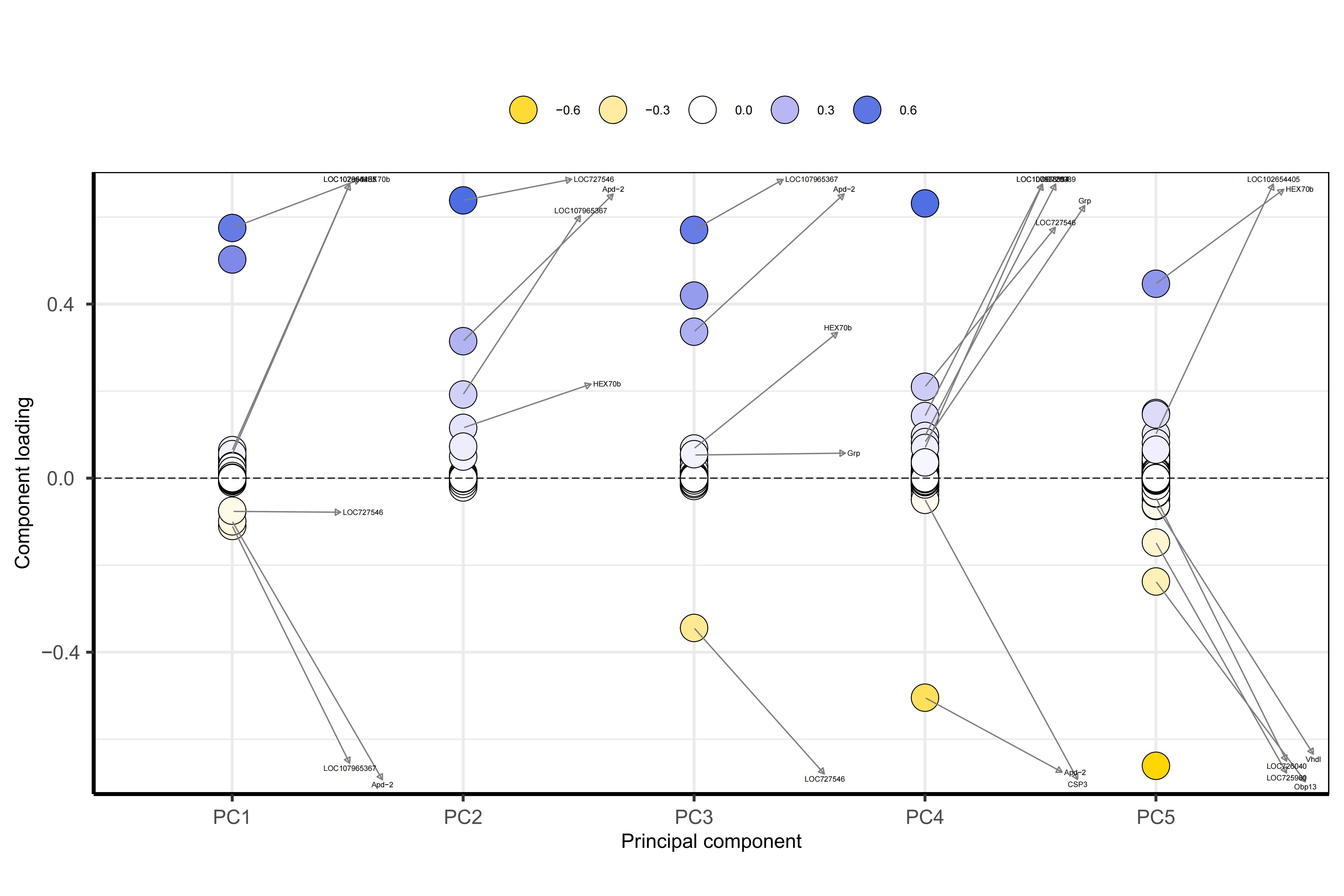

Supplement: Supplementary file 5 [file Image1.jpeg]

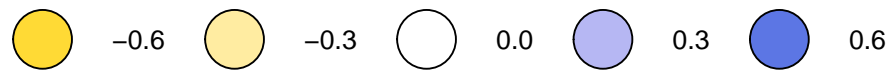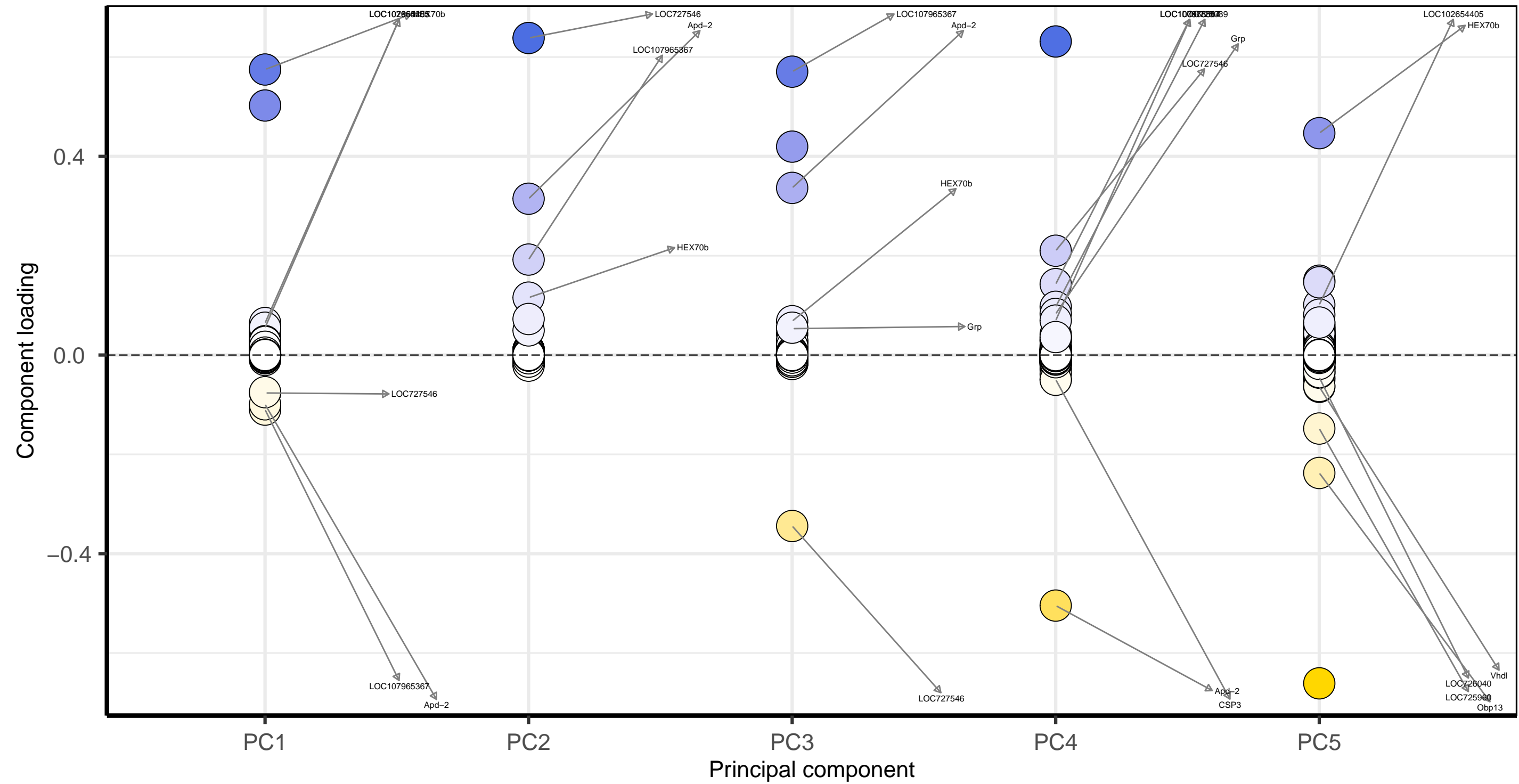

Supplement: Supplementary file 9 [file DataSheet1.pdf]
